# Evaluation of Applied Kinesiology meridian techniques by means of surface electromyography (sEMG): demonstration of the regulatory influence of antique acupuncture points

**DOI:** 10.1186/1749-8546-4-9

**Published:** 2009-05-29

**Authors:** Roy Moncayo, Helga Moncayo

**Affiliations:** 1 WOMED, Karl-Kapferer-Strasse 5, 6020 Innsbruck, Austria

## Abstract

**Background:**

The use of Applied Kinesiology techniques based on manual muscle tests relies on the relationship between muscles and acupuncture meridians. Applied Kinesiology detects body dysfunctions based on changes in muscle tone. Muscle tonification or inhibition within the test setting can be achieved with selected acupoints. These acupoints belong to either the same meridian or related meridians. The aim of this study is to analyze muscle sedation and tonification by means of surface electromyography.

**Methods:**

Manual muscle tests were carried out using standard Applied Kinesiology (AK) techniques. The investigation included basic AK procedures such as sedation and tonification with specific acupoints. The sedation and tonification acupoints were selected from related meridians according to the Five Elements. The tonification effect of these acupoints was also tested while interfering effects were induced by manual stimulation of scars. The effects of selective neural therapy, i.e. individually tested and selected anesthetic agent, for the treatment of scars were also studied. The characteristics of muscle action were documented by surface electromyographys (sEMG).

**Results:**

The sEMG data showed a diminution of signal intensity when sedation was used. Graded sedation resulted in a graded diminution of signal amplitude. Graded increase in signal amplitude was observed when antique acupuncture points were used for tonification. The tactile stretch stimulus of scars localized in meridian-independent places produced diminution of signal intensity on a reference muscle, similar to sedation. These changes, however, were not corrected by tonification acupoints. Correction of these interferences was achieved by lesion specific neural therapy with local anesthetics.

**Conclusion:**

We demonstrated the central working principles, i.e. sedation and tonification, of Applied Kinesiology through the use of specific acupoints that have an influence on manual muscle tests. Sedation decreases RMS signal in sEMG, whereas tonification increases it. Interfering stimuli from scars were corrected by selective neural therapy.

## Background

The basic examination technique used in Applied Kinesiology (AK) is a manual muscle test [1]. Applied Kinesiology is a system of evaluating body reactions to different stimuli that interact with the nervous system [2]. Recent reviews highlight historical, practical and methodological aspects of manual muscle testing [3-5]. During the development of AK [1], Goodheart described an association between meridians and individual muscles. The sedation and tonification acupoints of the corresponding meridian were used in a similar way as in acupuncture. These effects are based on the relations defined by the Five Elements (*wuxing*): Water (*shui*), Wood (*mu*), Fire (*huo*), Earth (*tu*) and Metal (*jin*) (Table 1). Tonification is based on the generating or mother-child relation, i.e. the *sheng *cycle [6]. Sedation is based on the inverse or child-mother relation [6]. In clinical practice, the use of these acupoints induces changes in the strength of the muscle being examined which can be perceived by the examiner as well as by the patient. When sedation is applied, muscle tone will diminish; when tonification is applied, muscle tone will increase.

**Table 1:** Mother-and-child Five Element acupoints of Chinese medicine and associated muscles according to Applied Kinesiology

Meridian	Element	Mother acupoint **~tonification**	Child acupoint **~sedation**	Associated muscles
Lung	Metal	LU9	LU5	Deltoids, Serratus anterior
Large intestine	Metal	LI11	LI2	Hamstrings, Tensor fascia lata
Stomach	Earth	ST41	ST45	Pectoralis major clavicular, Sternocleidomastoideus
Spleen	Earth	SP2	SP5	Latissimus dorsi
Heart	Fire	HT9	HT7	Subscapularis
Small intestine	Fire	SI3	SI8	Rectus abdominis, Rectus femoris
Urinary bladder	Water	UB67	UG65	Peroneus, Tibialis anterior
Kidney	Water	KI7	KI1	Iliopsoas
Pericardium	Fire	PC9	PC7	Gluteus medius, Gluteus maximus, Piriformis, Adductors
Triple heater	Fire	TH3	TH10	Teres Minor, Infraspinatus
Gall bladder	Wood	GB43	GB38	Popliteus
Liver	Wood	LV8	LV2	Pectoralis major sternal, Rhomboids

A further development of AK was made by Burtscher *et al. *[7] leading to a technique called AK meridian therapy (AKMT). While the name AKMT is similar to that of Goodheart [1] and Walther [2], Burtscher expands the regulatory possibilities of AK through the use of element acupoints of associated meridians according to the Five Elements [6] in order to achieve sedation or tonification (Table 2).

**Table 2:** Strategy for tonification or sedation based on Applied Kinesiology meridian therapy

	Within the same meridian		Acupoints from related meridians	
Tonification	Tonification acupoint = *Sheng *cycle	Support Grandchild-grandparent	Element acupoint of the generating meridian	Element acupoint of the grandchild meridian
Sedation	Sedation acupoint Child-mother	Control acupoint grandparent-grandchild	Element acupoint of the son meridian	Element acupoint of the grandparent

While there is abundant literature on the use of manual muscle tests for the evaluation of musculoskeletal disorders, only few studies have evaluated the effects of acupuncture on muscle function in clinical situations similar to those of AK. Costa and de Araujo [8] recently described the local effects of acupuncture on the tibialis anterior muscle. By needling the ST36 and SP9 acupoints, they observed a diminution of root mean square (RMS) signal of the tibialis anterior.

Since 2003, we have used the AKMT techniques for the evaluation of musculoskeletal diseases at our institute (WOMED, Innsbruck Austria), and used surface electromyography (sEMG) to investigate the functional correlations between manual muscle tests and AKMT points. The aim of this article is to report these sEMG data.

## Methods

### Subjects

Typical cases with an indicator muscle that presents a normal tone were selected. Both tonification and sedation were applied as needed. This evaluation included six adults without clinical evidence of disease. Four subjects were medical students and two were medical doctors. All gave informed consent. The first four subjects presented no surgical scars, while the last two had surgical scars. One had abdominal surgery for the correction of chryptorchidism, inguinal hernia (on the right side) and varicocele (on the left side). The other presented a T-shaped scar on the right shoulder (deltoideus muscle).

### AKMT examination – sedation and tonification

In this study, we tested the rectus femoris muscle only in order to limit the use of acupoints to those of the associated meridian, i.e. the Small Intestine (SI) meridian. The test procedure was as described by Walther [2]. Further clinical guidelines were recently provided by Schmitt and Cuthbert [4]. The test was carried out when the subject laid supine with the right leg flexed at the knee (90°). The AK test evaluated a transitory state called 'quality of resistance' [9], which required a coordinated start of the procedure where the examiner applies pressure on the tested muscle, while the subject attempted to maintain this 'resistance'. The state of resistance was recorded with superficial electromyography (sEMG). To demonstrate the principles of sedation and tonification of Applied Kinesiology Meridian Therapy (AKMT), we applied gentle digital pressure for about two seconds on specific acupoints of the SI meridian. Gentle pressure was the pressure that the examiner needed to feel the medium level pulse position [10]. According to the theory of the Five Elements [7], the tonification acupoint is SI3 (Wood) and the sedation acupoint is SI8 (Earth). Furthermore, sedation was achieved via the element acupoint of the son meridian (ST36) or grandparent meridian (BL66). The same principles apply to tonification procedures leading to the use of the element acupoint of the mother meridian (GB41) or grandchild meridian (LI1).

In the analysis of sedation, three subjects were studied. In the first subject, simple sedation was done via only the SI8 acupoint. In the second and third subjects, a sedation row was carried out via the SI8, ST36 and BL66 acupoints. In the tonification study, GB41 and LI1 acupoints were used. A rest interval of ten seconds between procedures was applied during the repeated tests of the rectus femoris. Preliminary sEMG studies showed that repeated measures had a variation of 5–8% of the resting RMS values. If the rest interval between tests is less than ten seconds, muscle weakness will be induced leading to a diminution of the RMS values. The patients with scars were examined under manipulation conditions. The initial muscle tone was recorded prior to scar manipulation, followed by a stretch stimulus of each scar (3–4 seconds) and repeated manual testing of the muscle together with sEMG recording.

### Clinical evaluation and treatment of scars

The clinical examination of the scars was performed with a dynamic challenge (stretching) based on AK techniques. In this setting a normotone muscle was used as an indicator. A positive test was when the dynamic stretch challenge had produced a change in the tonicity of the indicator muscle (either weakness or increased tone). The same challenge was repeated for the selection of the appropriate neural therapy (NT) agent which was held in the hand of the examiner during the challenge. In the evaluation of selective NT, the following anesthetic agents were considered: bupivacaine 0.25%, mepivacaine 0.5%, mepivacaine 2.0%, procaine 1.0%, lidocaine 0.5% and lidocaine 1%. The appropriate NT agent was one that eliminated the effect of the dynamic challenge. For hardened, hypertrophic scars, a local therapy with zinc oxide cream [11,12] was done prior to NT (Zinksalbe, Gall Pharma, Judenburg, Austria). The zinc cream was used 2–3 times per day for at least two weeks. The scars were treated with the cream with digital pressure applied along the axis of the scar as well as in a tangential orientation. Once the scars showed a softer consistence, the test for selective NT was completed.

### Surface electromyography (sEMG) analysis

For the sEMG analysis, the initial tone of the indicator muscle was recorded first. The influence of the acupoints mentioned above was tested with gentle digital pressure applied for two seconds on each acupoint and the sEMG recording repeated. sEMG was carried out with a 4-channel Bagnoli™ Desktop EMG System (Delsys, Boston, MA, USA). Data analysis was performed with the root mean square (RMS) calculated for each recording. Finally the data were reported as signal amplitude (EMGworks Analysis 3.6; Delsys, Boston, MA, USA).

## Results

**Figure 1:**
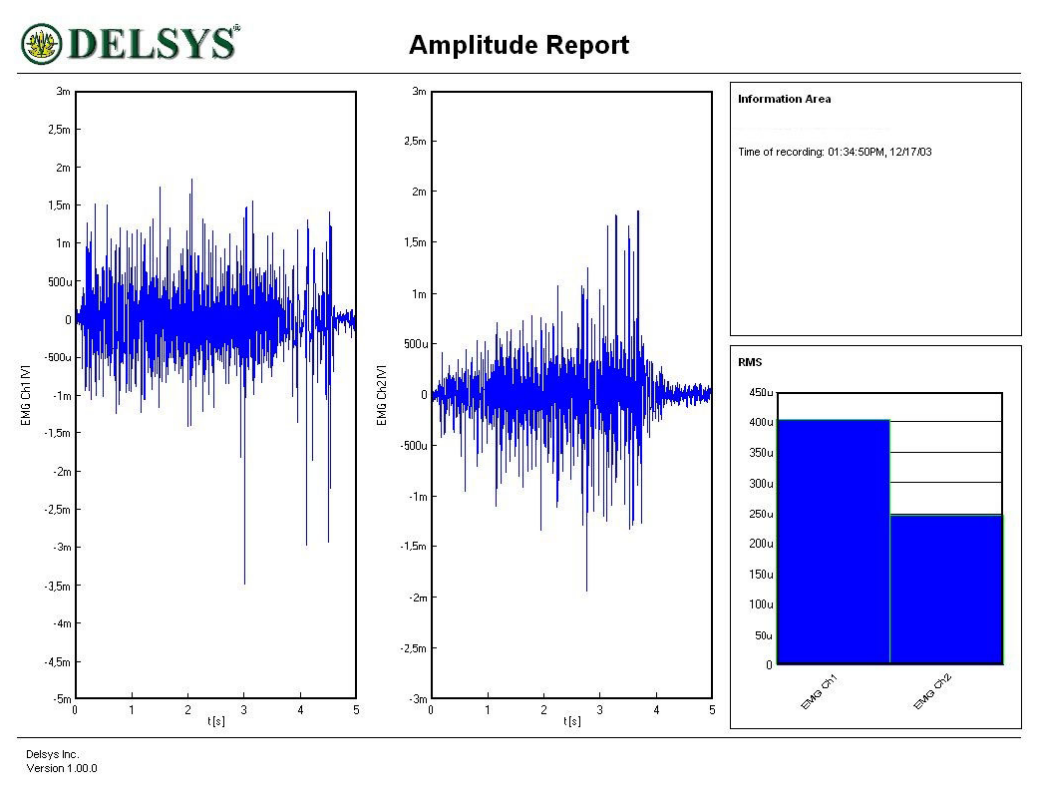
**Effect of the sedation acupoint SI8 on sEMG signal of the rectus femoris**. The signal amplitude during the initial muscle test was 400 μV; after sedation the amplitude decreased to 250 μv.

**Figure 2:**
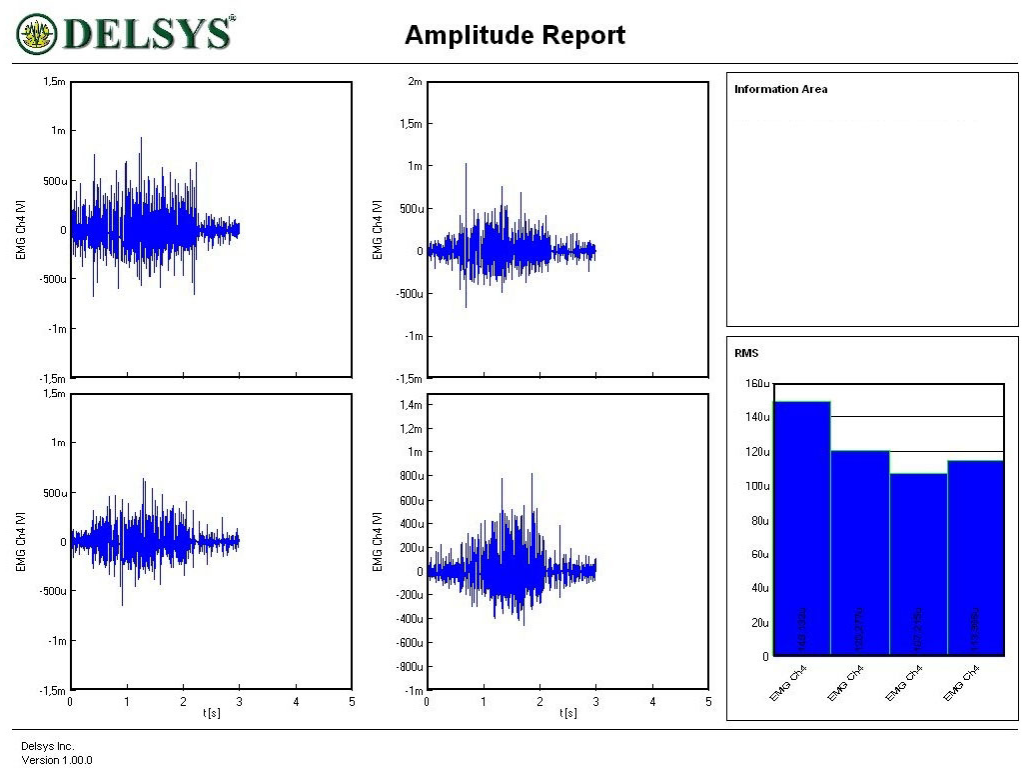
**Effect of the sedation acupoints SI8, ST36 and BL66 on the sEMG signal of the rectus femoris**. The signal amplitude during the initial muscle test was 150 μV; sequential use of sedation acupoints reduced the amplitude to 120, 110 and 114 μV.

**Figure 3:**
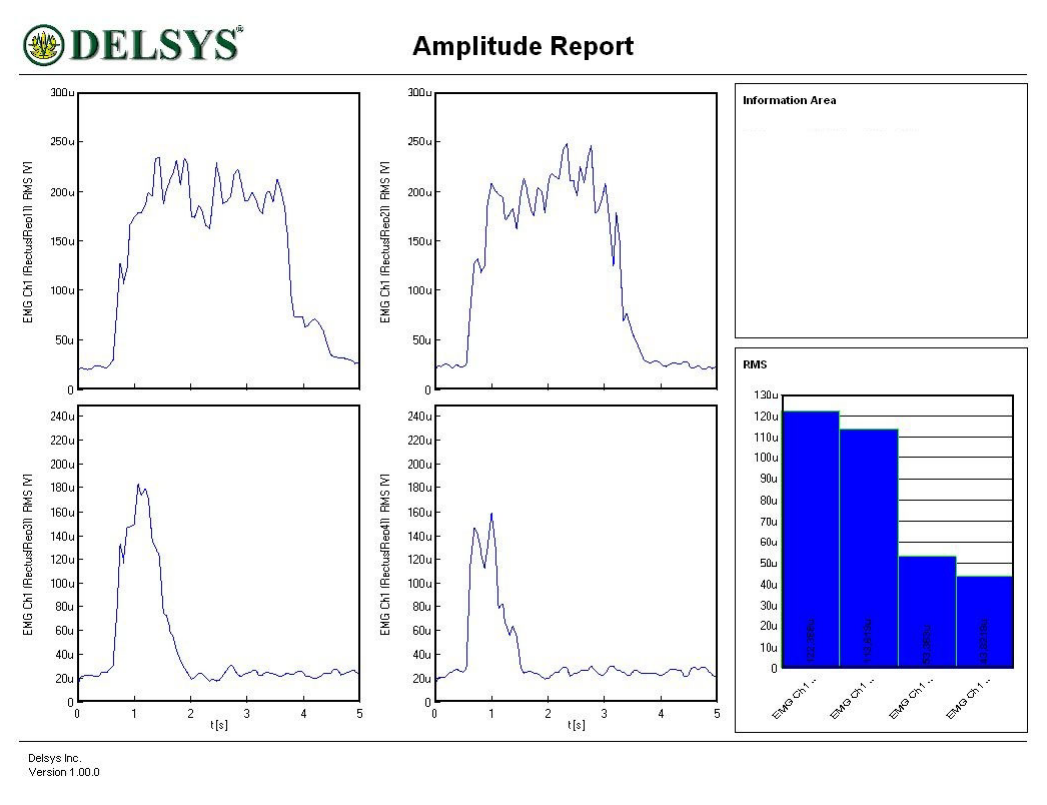
**Effect of the sedation acupoints SI8, ST36 and BL66 on the sEMG signal of the rectus femoris**. The signal amplitude during the initial muscle test was 120 μV; sequential use of sedation acupoints reduced the amplitude to 115, 50 and 40 μV.

**Figure 4:**
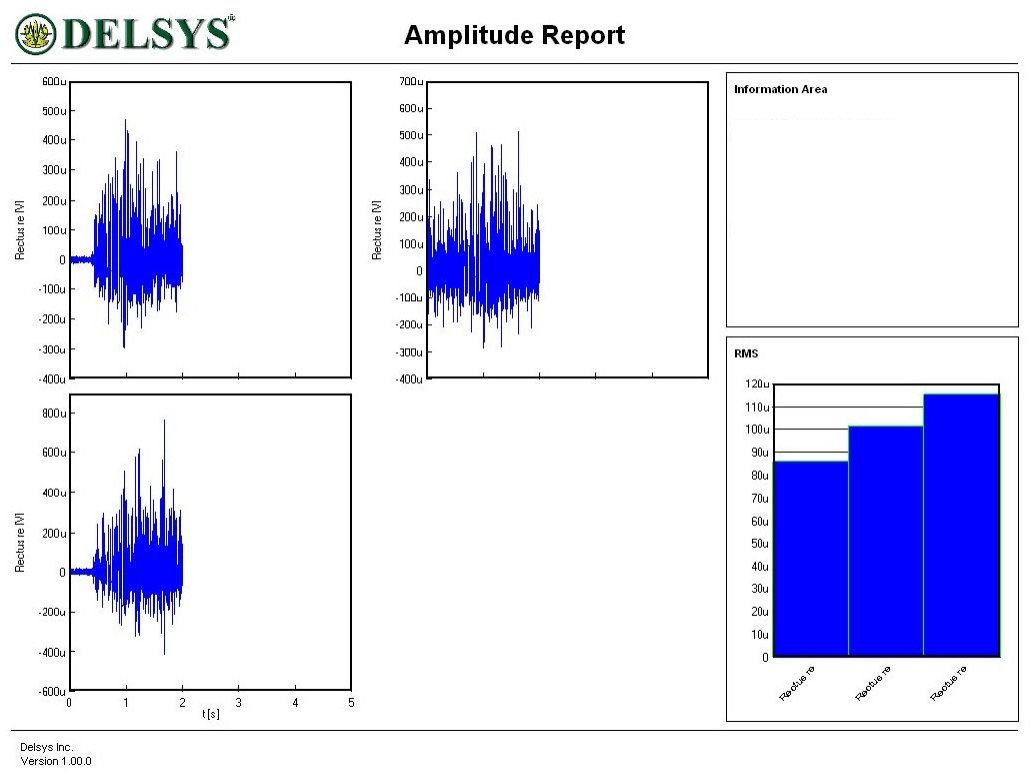
**Effect of the use of tonification acupoints GB41, and LI1 on the dynamics of the right rectus femoris**. The signal amplitude during the initial muscle test was 85 μV; sequential use of tonification acupoints increased the amplitude to 102 and 115 μV.

**Figure 5:**
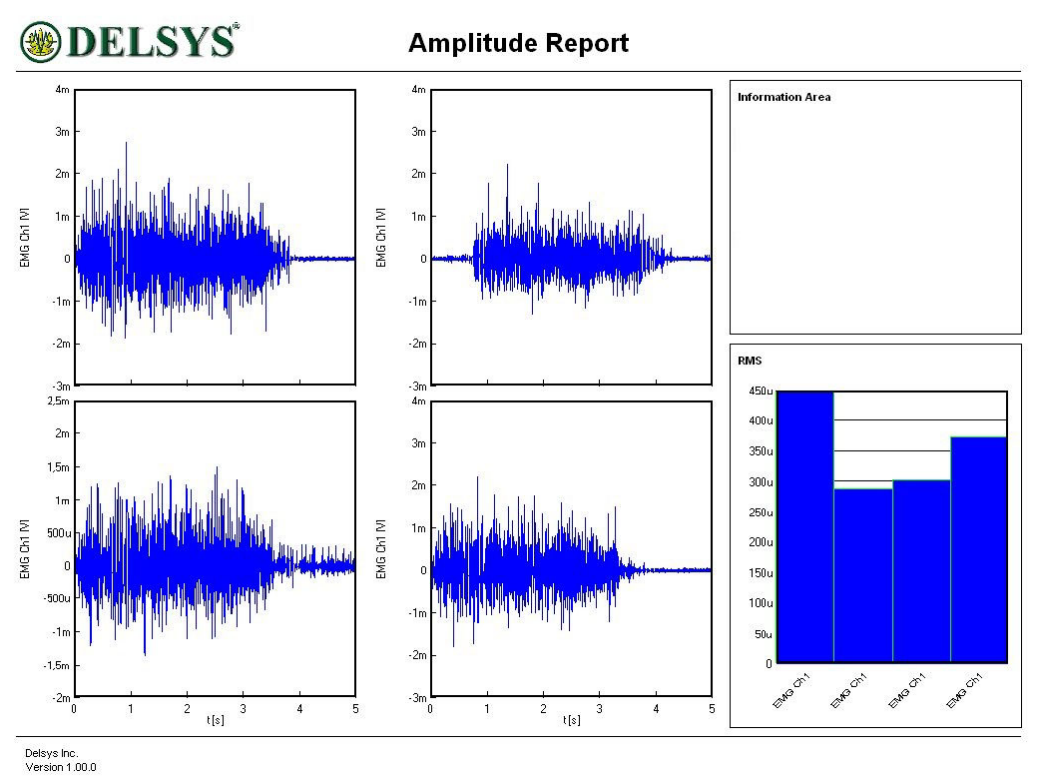
**Interfering – sedating effect of abdominal scar stimulation on the dynamics of the right rectus femoris**. The signal amplitude during the initial muscle test was 450 μV; sequential stretch stimulation of three different abdominal scars diminished the amplitude to 280, 300 and 370 μV respectively. This effect could not be reversed by tonification acupoints.

**Figure 6:**
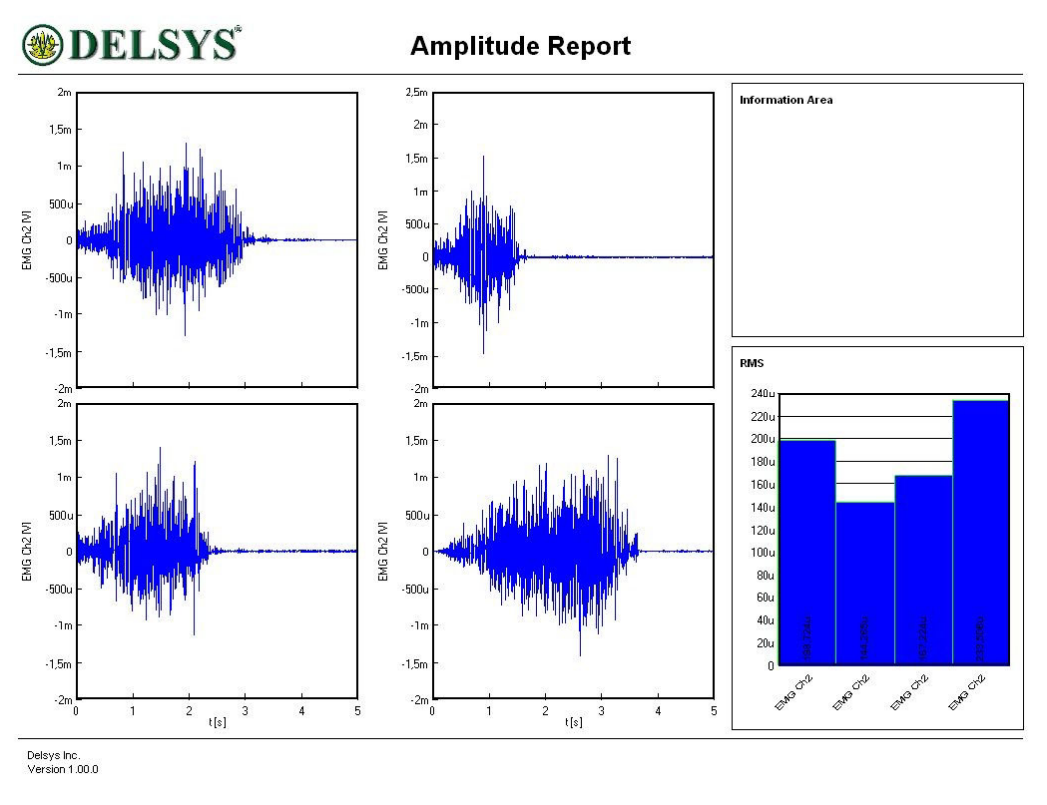
**Beneficial effect of selective NT on the dynamics of the right deltoideus**. The signal amplitude during the initial muscle test was 195 μV; sequential stretch stimulation of two different scars on the shoulder diminished the amplitude to 142 and 166 μV. This negative effect could be reversed selective NT. The resulting signal amplitude was higher (232 μV) than the initial value.

Figures 1, 2, 3, 4, 5 and 6 show amplitude graphs of the sEMG data of each of the selected procedures. Table 3 displays the acutal RMS data obtained in each procedure. The dimension of RMS signal change following sedation, tonification or scar manipulation was greater than the basal value for repeated measures at ten seconds interval (variation 5–8%). In each graph the right inset shows the initial muscle signal on the left-most column.

**Table 3:** Percentage change of the initial RMS values following either sedation or tonification based on AKMT.

Subject	Initial signal	Sedation		
	in μV	SI 8	ST 36	BL 66
1	400	62.5%		
2	150	80%	73.3%	76%
3	120	95.8%	41.7%	33.3%
				
	Initial signal	Tonification		
		GB 41	LI 1	
4	85	120%	135%	
				
	**Sedating interference through manipulation of three different abdominal scars**			
	Initial signal	Scar 1	Scar 2	Scar 3
5	450	62.2%	66.7%	82.2%
				
	**Therapeutic influence of neural therapy of scars**			
	Initial signal	Scar 1	Scar 2	Scar 1 after NT
6	185	76,8%	89,7%	125,4%

The sedation influence of scar manipulation is also shown. Repeated sEMG measures showed a variation of 5 to 8%.

The most common and basic situation in AK, i.e. the use of the sedation acupoint within the same meridian (SI8), led to a diminution of signal amplitude (Figure 1). A more graded pattern of response was observed when other sedating acupoints were used (Figures 2 and 3). Each subject showed, however, an individual response pattern. In the tonifying procedure, a constant increase in signal amplitude was recorded (Figure 4).

In subject 5, manual stretch stimulus of each of the three abdominal scars tested led to a diminution of signal intensity of the rectus femoris (Figure 5). Correction of this sedating effect could not be achieved by the use of tonification acupoints. In such situations, selective NT (a specific anesthetic agent for each scar) [13] was carried out as needed. In our routine work, mostly for treating hypertrophic scars, the three most commonly used agents are procaine, mepivacaine and lidocaine, in 34.8%, 30.4% and 26.1% of cases (*n *= 115) respectively (time period 2002–2005). The selective NT was carried out effectively in subject 6. Manipulation of the two shoulder scars produced initially a diminution of the RMS signal. Already ten minutes after NT, scar manipulation did not alter the muscle tone. Surprisingly, the 'new' basal RMS value improved compared to the initial basal value (Figure 6).

## Discussion

Caruso and Leisman described the AK test as one that evaluates a transitory state named 'quality of resistance' [9]. The sEMG analyses we did support this notion of change in the 'quality of resistance', i.e. diminution of RMS amplitude under sedation and the opposite during tonification. These effects can be achieved via acupoints of the same meridian or of associated meridians according to the theory of the Five Elements. The use of additional acupoints of tonification or sedation show slight differences in the absolute RMS signals; however the use of sEMG is not mandatory for clinical practice. Experienced AK practitioners can still rely on the good practice for conducting manual muscle tests [4]. In a similar study based on AK principles, Zampagni *et al. *[14] used a tailor-made device equipped with a load cell in order to evaluate the strength of the tensor fascia lata in different conditions. The changes in the recorded signals are in agreement with the principles of AK in relation to sedation and tonification.

It must be stressed that the conduct of manual muscle tests must follow good practice guidelines in order to obtain reliable results [4]. The combination of manual muscle testing and acupuncture principles results in a unique and simple examination procedure when compared with other methods such as electroacupuncture [15], laser acupuncture [16] or computer-based meridian diagnosis [17,18]. In clinical practice, changes of the 'quality of resistance' of the test muscle are also felt by the patient. Understanding the examination procedure adds a psychological dimension to the treatment [19]. Manual contact and interaction has the potential of improving the patient-doctor communication [20].

According to a recent study by Costa and de Araujo, functional changes of the tibialis anterior muscle can be induced via needling ST36 and SP9 acupoints [8]. We would like to offer an acupuncture-based interpretation of their results. According to AK, the tibialis anterior muscle corresponds to the Bladder meridian. The sedating effect of ST36 corresponds to the grandparent relation between the ST and BL meridians. The sedation effect seen when SP9 was needled could be explained by the inner-outer relation that exists between these two acupoints. According to AKMT [7], the needling of inner-outer related meridians will reinforce the complementary partner, in this case ST36, which then shows effects corresponding to the grandparent relation between ST and BL. We feel that this explanation based on principles of Chinese medicine is more appealing than the term 'reflex loop' used by Castro and de Araujo in their publication.

### The role of scars

Some studies have suggested an intimate relation between muscular and fascial structures [21,22]. Scars would alter the integrity of these structures [23]. The skin and the underlying anatomical structures require a smoothly functioning sliding system [24,25], and at a deeper level, skin contact with the underlying fascia is a central event of structural and functional integrity [26]. The present study provides an example of the interaction of scars with the manual muscle test. In our clinical experience, we observe that the use of zinc oxide with selective NT can overcome this situation. The relevance of this procedure is that of eliminating interfering influences on superficial force transmission through the fasciae [24,25,27,28].

## Conclusion

Traditional acupuncture concepts of tonification and sedation are applicable to clinical studies based on manual muscle techniques of AK. Tonification leads to an improved function of any meridian while sedation reduces the excess. In terms of muscle signals derived from sEMG, signals increase in tonification and decrease in sedation. Surgical scars can cause interferences in manual muscle tests as well as in muscle tone and require treatment by means of selective NT.

## Abbreviations

AK: Applied Kinesiology; AKMT: Applied Kinesiology meridian therapy; NT: neural therapy; RMS: root-mean-square; sEMG: surface electromyography.

## Competing interests

The authors declare that they have no competing interests.

## Authors' contributions

Both RM and HM designed the study and wrote the manuscript. RM performed the sEMG studies. Both authors read and approved the final version of the manuscript.
